# Immune Evasion in Stem Cell-Based Diabetes Therapy—Current Strategies and Their Application in Clinical Trials

**DOI:** 10.3390/biomedicines13020383

**Published:** 2025-02-06

**Authors:** Razik Bin Abdul Mu-u-min, Abdoulaye Diane, Asma Allouch, Heba Hussain Al-Siddiqi

**Affiliations:** 1 Diabetes Research Center, Qatar Biomedical Research Institute (QBRI), Hamad Bin Khalifa University (HBKU), Qatar Foundation (QF), Doha P.O. Box 34110, Qatar; adiane@hbku.edu.qa (A.D.); halsiddiqi@hbku.edu.qa (H.H.A.-S.); 2 College of Health and Life Sciences (CHLS), Hamad Bin Khalifa University (HBKU), Qatar Foundation (QF), Doha P.O. Box 34110, Qatar; aallouch@hbku.edu.qa

**Keywords:** β-cells, diabetes, immune evasion, stem cells, transplantation

## Abstract

**Background/Objectives**: Human pancreatic islet transplantation shows promise for long-term glycemic control in diabetes patients. A shortage of healthy donors and the need for continuous immunosuppressive therapy complicates this. Enhancing our understanding of the immune tolerance mechanisms related to graft rejection is crucial to generate safer transplantation strategies. This review will examine advancements in immune protection strategies for stem cell-derived islet therapy and discuss key clinical trials involving stem cell-derived β-cells and their protective strategies against the host immune system. **Methods**: A comprehensive literature search was performed on peer-reviewed publications on Google Scholar, Pubmed, and Scopus up to September 2024 to extract relevant studies on the various strategies of immune evasion of stem cell-derived β-cells in humans. The literature search was extended to assimilate all relevant clinical studies wherein stem cell-derived β-cells are transplanted to treat diabetes. **Results**: Our analysis highlighted the importance of human pluripotent stem cells (hPSCs) as a potentially unlimited source of insulin-producing β-cells. These cells can be transplanted as an effective source of insulin in diabetes patients if they can be protected against the host immune system. Various strategies of immune protection, such as encapsulation and genetic manipulation, are currently being studied and clinically tested. **Conclusions**: Investigating immune tolerance in hPSC-derived islets may help achieve a cure for diabetes without relying on exogenous insulin. Although reports of clinical trials show promise in reducing insulin dependency in patients, their safety and efficacy needs to be further studied to promote their use as a long-term solution to cure diabetes.

## 1. Introduction

Diabetes mellitus constitutes a group of chronic and metabolic disorders characterized by prolonged elevated blood glucose levels. The primary mechanism attributed to the onset and impact of the disease is insulin deficiency, resulting in impaired glucose metabolism. The International Diabetes Federation (IDF) reported that, as of 2021, 537 million adults are living with diabetes, a number that is projected to rise to 783 million by 2045. Additionally, 6.7 million deaths were attributed to diabetes-related comorbidities (www.idf.org). The American Diabetes Association (ADA) classifies diabetes into two main categories: Type 1 diabetes (T1DM) and Type 2 diabetes (T2DM) [1]. T1DM stems from the immune-mediated destruction of pancreatic β-cells, causing a near-complete deficiency in insulin production. While T1DM constitutes only 5–10% of diabetes cases, it represents 80–90% of cases in children and adolescents [2,3,4]. Conversely, T2DM occurs when individuals experience insulin resistance and a relative insulin deficiency due to β-cell dysfunction, accounting for 90–95% of all diabetes cases [5]. Other diabetes types encompass gestational diabetes mellitus (GDM), neonatal diabetes, and maturity-onset diabetes of the young (MODY) [6]. It is widely recognized that all forms of diabetes share a commonality in dysfunctional pancreatic β cells negatively affecting insulin secretion. Diabetes is associated with additional health burdens, manifesting in macrovascular complications such as stroke, peripheral artery disease, coronary heart diseases, and myocardial infarctions, as well as microvascular complications like neuropathy, retinopathy, and nephropathy. These complications lead to a reduced quality of life and premature death [7]. The treatment and management of T2DM involves a multifaceted approach combining lifestyle adjustments such as dietary habits, physical activity, sufficient sleep, and pharmacological interventions [8,9,10,11,12]. The current approach to treating T1DM involves managing glycemia through daily insulin supplementation, administered either via insulin injections or insulin pumps equipped with integrated glucose monitors [13,14]. Despite being lifesaving, these invasive treatment methods have limitations, often leading to acute hypoglycemia, which, in turn, can contribute to heart and kidney failures. An alternative treatment for T1DM is cadaveric islet transplantation using the Edmonton protocol. This approach offers the potential for temporary exogenous insulin independence [15]. However, its feasibility is increasingly hindered for the growing T1DM patient population due to factors such as a shortage of donors, and, importantly, the potential risk of graft rejection, necessitating lifelong use of immunosuppressive drugs [16,17].

### 1.1. Graft Rejection/Immune Protection in Islet Transplantation

Graft rejection or progressive loss of islet function in transplanted pancreatic islets occurs due to autoimmune and alloimmune reactions. Graft rejection occurs primarily through “direct” and “indirect” pathways in the host system. The direct pathway is mediated by the host (recipient) T cells, as T cells are activated via T cell receptor (TCR) recognition of antigenic peptides presented by major histocompatibility complexes (MHCs) expressed on the donor cells. This results in a direct attack by recipient T cells on the transplanted cells, leading to graft destruction. In the indirect pathway of graft rejection, donor specific MHCs are recognized by recipient antigen presenting cells (APC), such as B cells, macrophages, and dendritic cells, and broken down into fragments which are then presented on the recipient APCs, thus triggering the recipient T cells. The rejection of transplanted cells can be due to either direct or indirect pathways and could work in cohorts to effect graft rejection [18,19,20,21,22]. Treatment strategies designed to circumvent graft rejection generally involve an immunosuppression program. In transplantation attempts, notably in the 1990s, graft rejection was prevented by treating the patients with an induction of immunosuppressive agent targeting T lymphocytes, followed by a three-pronged immunosuppression strategy consisting of a calcineurin inhibitor, a DNA antimetabolite, and steroids. However, all these agents demonstrated, in varying degrees, β-cell toxicity or diabetogenicity [23]. The pioneering work of the Edmonton protocol introduced a steroid-free regimen that improved graft survival and insulin independence in T1DM patients. The treatment consisted of the anti-interleukin-2 receptor antibody ‘daclizumab’ to inhibit inflammation, the mammalian target of rapamycin inhibitor ‘sirolimus’ to deplete T cells, and the calcineurin inhibitor ‘tacrolimus’ to block T cell proliferation [15]. Although patients were able to sustain insulin independence for half a decade, immunosuppression-mediated side effects were prominent [24]. Multiple combinations of immunosuppressive treatment strategies were developed subsequently, with varying degrees of success. A study used an antibody-based approach where anti-thymocyte globulin, daclizumab, and etanercept were used for the induction phase; followed by mycophenolate mofetil to inhibit T cell proliferation; and lymphocyte adhesion molecules, sirolimus, and either no or low-dose tacrolimus for maintenance. Patients administered with this treatment regimen managed to retain insulin independence for over a year [25]. Other strategies of immunosuppression involved using hOKT3γ, an anti-CD3 antibody, to reduce T cell expression for the induction phase, followed by sirolimus- and tacrolimus-based immunosuppression [26]. Studies have also highlighted the success of an effective calcineurin inhibitor-free immunosuppression protocol using the co-stimulation blocker belatacept, and patients were able to achieve long-term insulin independence [27]. The major pitfalls in most immunosuppressive regimens include β-cell endoplasmic reticulum (ER) stress, faulty revascularization of grafts, impaired β-cell replication, impaired glucose-stimulated insulin secretion (GSIS), and reduced insulin sensitivity [28,29,30,31]. Moreover, patients are at risk of developing infections and tumors as immunosuppression compromises their immune system [22].

### 1.2. The Promise of Stem Cells in Regenerative Cell Therapy in Diabetic Patients

**Figure 1:**
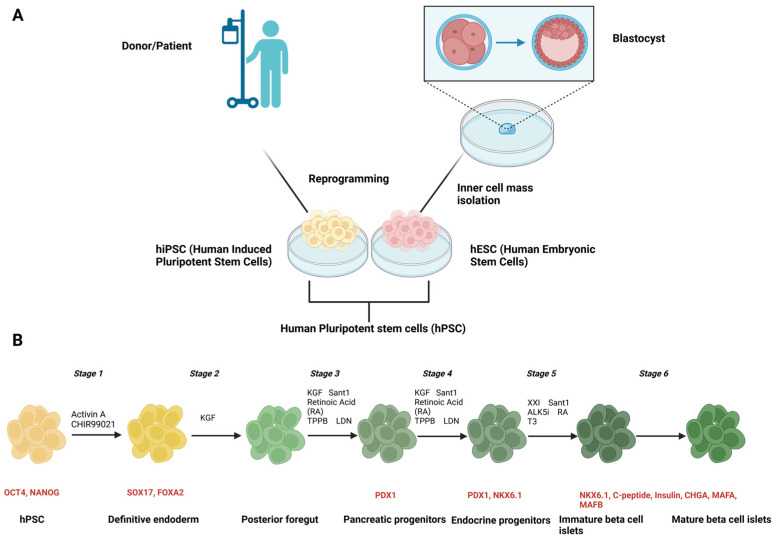
Schematic of the generation of stem cell-derived pancreatic β-cell islets. (**A**) hESCs are extracted from the inner cell mass of blastocyst stage of developing embryo and cultured to proliferate indefinitely. hiPSCs are generated through direct reprogramming of somatic cells from a donor to pluripotent stem cells. (**B**) A general protocol outlining the differentiation stages of the generation of pancreatic β-cell islets from hPSCs. The resulting cell stages are labelled in black and the quality control markers to track the efficiency (by flow cytometry or immunocytochemistry) of differentiation at each stage are labelled in red. This illustration was created with “BioRender.com”.

Advancements in regenerative medicine have recently highlighted the development of transplantable functional pancreatic β-cells derived from human pluripotent stem cells (hPSCs), both human embryonic stem cells (hESCs) and induced pluripotent stem cells (hiPSCs), as a potential diabetes treatment. These patient-specific-derived β-cells, also referred to as autologous PSC-derived β-cells, offer a promising solution to address challenges like insufficient islet availability by serving as an unlimited source of β-cells for diabetes therapy and graft rejection by using patient-specific stem cell lines. Additionally, patient-specific stem cell-derived β-cells in vitro are being used to study diabetes-related mutations, including inherited monogenic diabetes, and explore disease progression [32,33]. Efforts are underway across the world to differentiate hPSCs efficiently and reproducibly into insulin-producing β-cells using multistage directed differentiated protocols (Figure 1). Directed differentiation of stem cells attempts to mimic the embryonic development of pancreatic β-cells by emulating growth stages through the sequential addition of small molecules and growth factors. The success of these protocols is typically gauged by how closely the resulting cells resemble their in vivo counterparts in terms of maturity and functional efficiency. Over the years, numerous research laboratories have established protocols featuring optimized culture conditions, differentiation media, and small molecules, and these protocols are applicable to multiple hPSC cell lines, leading to an improved efficiency in differentiation and an enhanced functionality of β-cells [34,35,36]. The transplantation of hPSC-derived β-cell islet-like clusters into mice has been shown to improve maturation and functionality, thereby providing evidence for the possibility of using them for human transplantation [37].

In this review, we briefly present various strategies devised to overcome immune rejection following transplantation, with particular emphasis on the promise and progress of using hPSCs to achieve this goal. An insight is provided into various clinical trials that employ stem cell-derived pancreatic progenitors/beta-like cells for transplantation and their outcome.

## 2. Immune Protection Using Encapsulation

Cell encapsulation has been considered an attractive addendum to the current transplantation techniques to avoid graft rejection post-transplantation by bypassing the need for an immunosuppressive drugs regimen. Transplanted cells are encased by semi-permeable biomaterials that block immune cells’ entry while being permeable to oxygen, nutrients, and the diffusion of metabolites. The two types of encapsulation strategies are microencapsulation, wherein a few cells are encased by microspheres of less than 1000 µm, and macroencapsulation, wherein millions of cells are encased in macroscopic polymeric devices [38]. The ideal islet encapsulation device design requires several considerations such as the capsule size, mode of surgery, and implantation site, among others [39].

### 2.1. Microencapsulation

The most used material for microencapsulation is alginate, a naturally occurring polysaccharide found in brown algae. The main components of alginate are the liner β-D-mannuronate and α-L-guluronate. Microcapsules are generated by crosslinking the polymer with divalent cations (e.g.,: calcium and barium). This forms a gel matrix suitable for encapsulating cells. The smaller size of the microcapsules as compared to macrocapsules provides them with a high surface-to-volume ratio that improves the diffusion of oxygen and other agents that are essential for β-cell survival [38,40]. Key alginate microcapsule factors, such as stability, pore size, immunogenicity, and cell-binding domains, can be manipulated by combining alginate with various compounds such as collagen, poly-L-lysine, pluronic F127, and immunomodulators such as dexamethasone and stromal cell-derived factor 1α (CXCL12) [41,42,43,44]. Studies have experimented with microencapsulation of β-cells followed by their transplantation. A 2016 study implanted hPSC-derived β-cells encased in barium–triazole–thiomorpholine dioxide alginate microcapsules into STZ-induced diabetic mice without immunosuppression. The implanted β-cells showed glucose responsiveness for 174 days, after which the retrieved implant still contained viable insulin producing cells [45]. Another study used an innovative technique which involved incorporating CXCL12 into sodium alginate microcapsules. hESC-derived β-cells encapsulated in these microcapsules showed enhanced insulin secretion in diabetic mice. Additionally, these cells were able to evade the foreign body response of cells wherein implanted materials are affected by fibroblast recruitment followed by collagen deposition around the implants, thereby affecting cell survival [46]. The cells were able to regulate hyperglycemia and maintain functionality for over 150 days without immunosuppression [47]. Microencapsulation isolates the cells from the host system with thick barriers that hinder the efficient diffusion of oxygen and essential metabolites, and could lead to delayed GSIS and islet necrosis due to a lack of oxygen supply [48,49]. A strategy devised to alleviate this issue is the encapsulation of cells in a conformal coating which has a much-reduced thickness in the order of tens of micrometers. Enhanced diffusion can be permitted in this setup while maintaining a barrier to the host immune system. A study created a polyethyleneglycol-based conformal coating to encapsulate human stem cells-derived islets which were then transplanted into diabetic non-obese diabetic/severe combined immunodeficiency (NOD-SCID) mice. The transplanted cells were able to reverse diabetes and maintain normal euglycemia for more than 80 days [50].

### 2.2. Macroencapsulation

In macroencapsulation, a large number of cells are generally encased in a perforated pouch or a large hydrogel over 1 mm long. Macroencapsulation devices provide good chemical and mechanical stability, in addition to the advantage of being retrievable post-transplantation in case of failure or malignant alteration of transplanted cells. Macroencapsulation techniques consist of hydrogel scaffold-based devices, membrane-controlled release systems, or microneedle array patches [51,52]. Several studies over the past decade have attempted to generate potential therapeutic applications of macroencapsulation devices combined with stem cell-derived β-cells. Earlier studies used a planar pouch-like encapsulation device using a bilaminar polytetrafluoroethylene membrane system (TheraCyte) which showed promising immunoprotection in transplanted cells in mice and primates [53,54,55]. The technology was later applied to hESC-derived pancreatic progenitors which were transplanted into mice. The cells achieved maturation within the macroencapsulation device and promoted insulin production with no change in biomass within the capsule for up to 150 days, suggesting high immunoprotection [55]. A 2017 study created a flexible and durable polycaprolactone-based 10 µm thick nanoporous thin-film cell-encapsulation device capable of neovascularization formation with minimal foreign body response and teratoma confinement, which increased the safety of the use of stem cell-derived β-cells. The membrane was also shown to exclude proinflammatory cytokines while promoting glucose and insulin exchange. The transplantation of the device encasing hESC-derived β-cells into immunocompetent mice showed long term biocompatibility as glucose responsiveness and cell viability were seen 6 months post-transplantation [56]. A recent study created a durable zwitterionically modified alginate hydrogel capable of evading foreign body responses. Stem cell-derived β-cells were encased in these macrocapsules and inserted into diabetic mice, which exhibited significantly better glucose clearance for over 230 days. Due to improved biocompatibility, mass transfer, and low fibrotic reactions, the device was capable of long-term cell engraftment [57]. Conventional macroencapsulation devices suffer from slow GSIS due to their reliance on diffusion of oxygen and metabolites. A recent study designed a convection-enhanced macroencapsulation device that enabled improved nutrient transfer across the device membrane, enabling a near 10-fold higher cell loading capacity. The polytetrafluoroethylene-based membrane chamber encased stem cell-derived β-cells suspended in matrigel, which enabled a homogenous cell distribution, prevention of aggregates, and lowered the shear stress during loading. Transplantation of the device into immunocompetent hyperglycemic rats demonstrated early improvement in hyperglycemia, improved GSIS, and reduced fibrosis [58]. An important consideration for the creation of micro and macroencapsulation devices is their ability to overcome a foreign body response. Various techniques have been adopted to manage a foreign body response evoked by encapsulation devices. These generally involve altering the device’s surface chemistry (e.g., charge, composition, hydrophobicity), coating the device to alter its topography, and manipulating its shape and porosity [59]. Although advances have been made in encapsulation technologies, efficient oxygenation still remains a challenge for the long-term survival of the grafts due to the lack of vascularization of intraislet capillaries as compared to native islets. Recent strategies devised the use of encapsulation materials that promote graft revascularization. Encapsulation devices with direct vascularization developed by ViaCyte Inc. and Sernova are undergoing clinical trials to evaluate their safety and efficacy. The disadvantage of such devices is the loss of immunoprotection for grafts and the requirement of a systemic immunosuppression regimen to prevent rejection.

Several clinical trials over the past decade have studied the transplantation of hPSC-derived pancreatic progenitor/β-cells protected by various encapsulation devices. A summary of relevant clinical trials and their outcomes is outlined in a later section of this review.

## 3. Genetically Modified hiPSC-Derived β-Cells for Immune Evasion

hiPSC technology holds a great promise in precision medicine powered by personalized treatment designed using one’s own somatic cells such as peripheral blood cells. The key advantage of using this technology is the production of patient-specific cells designed for autologous transplantation, thereby bypassing immune-driven organ rejection, and improving cell survival after transplantation [60]. Currently, the cost and time required to produce autologous hiPSC-derived β-cells hinder the effectiveness of this treatment strategy, although inroads are being made to better streamline the technology. Recognition of the cell surface markers MHC, also known as human leukocyte antigens (HLAs), by T cells is one of the most important criteria of allogeneic transplant rejection in humans. The genes encoding HLAs are classified into class I, comprising HLA-A, -B, -C, -D, -E, -F and -G; class II, comprising HLA-DR, -DP, -DQ, -DM, -DN, and -DO; and class III, which encodes for proteins of the complement system and the TNF family genes. HLA-A, -B, -DR, -DP, and -DQ are believed to contribute the most to immune rejection. Attempts have been made to generate HLA homozygous hiPSC lines which can potentially be used to transplant into patients to prevent HLA-mediated allorejection. hiPSCs from donors with homozygous HLA-A, -B, and -DR have been selectively generated and cryopreserved for their potential future use to generate β-cells which could be less reactive to the host immune system. However, a much larger scale study needs to be performed to account for genetic diversity in the population to create universal hiPSC lines that can be used globally. Also, this is not a complete solution to dealing with immune rejection, since the HLA haplotypes HLA-DR4-DQ8 and HLA-DR3-DQ2 have been implicated in immune responses wherein insulin is presented as antigens to activated T cells [61,62,63]. MHC expression in stem cells is generally low and the cells are therefore protected from immune responses. However, differentiation stimulates MHC expression and renders the cells vulnerable to immune system [64]. Stem cell-derived β-cells generally express class I antigens which are upregulated under inflammatory stress. A study reported that stem cell-derived β-cells largely express HLA-C, while native β-cells express all class I MHCs. The authors suggested that the difference in the cell properties with respect to MHC expression might be due to the immaturity of stem cell-derived β-cells as compared to native β-cells [65]. Another important candidate for immune response regulation is the immune checkpoint inhibitor programmed death-ligand 1 (PD-L1), which is upregulated as a protection agent in response to pro-inflammatory cytokines. Gene manipulation strategies to circumvent immune responses in transplanted cells are discussed below.

Genome editing, a powerful tool to create gene knockouts and knock-ins, is finding stronger footing in improving our understanding of the underlying genetic mechanisms of diseases. Genome editing tools work under the premise where a nuclease identifies a target sequence, induces a double-stranded DNA break (DSB), and activates endogenous cellular DNA repair mechanisms such as homologous recombination or non-homologous end-joining. In the field of diabetic research, genome editing has already been used to create models of diabetes subtypes from hESCs and hiPSCs [66,67]. Commonly used gene editing systems include zinc finger nucleases (ZFN), transcription activator-like effector nuclease (TALEN), and the promising Clustered Regularly Interspaced-Short Palindromic Repeats (CRISPR)/Cas9 technology. ZFNs are based on zinc finger proteins, a cohort of transcription factors fused on a restriction endonuclease *Fok*I that effects gene edits [68]. TALENs take advantage of the proteins ‘transcription activator-like effectors (TALEs)’ to provide high specificity to gene editing, while maintaining a lower cytotoxicity and simpler design structure as compared to ZFNs [67,69]. The CRISPR/Cas9 system utilizes short noncoding guide RNAs (sgRNA) to identify specific target DNA sequences and is combined with the enzyme CRISPR-associated protein 9 (Cas9) to effect gene cleavage [70].

Based on our current understanding of the immune responses on hPSC-derived β-cells post-transplantation, strategies can be developed to genetically engineer functional hPSC-derived β-cells which are immune evasive and thereby improve β-cell survival and functionality in diabetes patients. One approach for genetically modifying hPSC-derived β-cells is to prevent HLA expression and therefore avoid T cell responses. Beta-2 microglobulin (B2M) is responsible for the proper folding and cell surface expression of HLA class I proteins and therefore its deletion would abolish HLA class I expression, as demonstrated by a study. In this study, a B2M gene knockout (KO) cell line was generated using CRISPR-Cas9 in hPSCs and showed that wildtype hPSC-derived β-cells induced higher levels of T cell activation as compared to the B2M knockout model [65]. In another study, CRISPR-Cas9 was used to disrupt B2M gene in hiPSCs-derived from T1DM patients. A co-culture of PBMCs with wildtype beta-like cells and HLA class I deficient beta-like cells showed a reduction in the expression of immune markers CD25 and CD69 in autologous CD8^+^ T cells, providing further evidence that HLA expression disruption can decrease immune responses [71]. Although preventing HLA class I antigens can prevent T cell-mediated immune responses, according to the “missing self” hypothesis, in the absence of all HLA-presenting antigens, natural killer cell-mediated lysis will be activated due to the lack of inhibitory signals on the target cells [72]. This effect was reported in studies which target-removed B2M in hPSCs, as cells became vulnerable in varying degrees to natural killer cell-mediated immune responses [73,74]. However, the targeted inactivation of HLA-A, -B, and -C genes while maintaining HLA-E expression alone, or in cohorts with HLA-G, CD47, and PD-L1, has proven to protect hPSCs from natural killer cell responses [75,76,77].

Another avenue for genetically engineering stem cells to evade immune responses is through PD-L1 overexpression. Cancer cells are known to evade T cell rejection by expressing the immune checkpoint molecule PD-L1, related to the T cell inhibitory receptor programmed death 1 (PD-1) [78]. Previous studies have shown that PD-L1 expression in islets protects against immune responses in the transplantation of syngeneic islets into diabetic recipients. PD-L1 also inhibited self-reactive CD4^+^ T cell-mediated tissue destruction and effector cytokine production [79,80]. Another study highlighted the importance of PD-L1 with respect to islet transplantation. The authors noticed that though a PD-L1 deficiency in donor hearts does not evoke immune rejection, PD-L1-deficient islets heightened allograft rejection, thereby emphasizing the importance of PD-L1 in islet function and immune responses. The transplantation of islets from PD-L1-deficient mice into STZ-induced diabetic mice induced graft rejection. They attributed the islet rejection to enhanced T cell activation and inflammatory cell infiltration [81]. Studies have explored the idea of overexpression of PD-L1 in stem cells to protect stem cell-derived islets from immune rejection. A study used CRISPR technology to overexpress PD-L1 stem cells and reported that stem cell-derived functional beta-like cells were partially protected from T cell responses [82]. A more recent study generated functional beta-like islets from hiPSCs, which were genetically modified using a lentiviral system to overexpress PD-L1. The transplantation of these stem cell-derived islet-like cells restored glycemic control in immune competent diabetic mice and maintained glucose homeostasis for over 50 days when compared to islet-like cells which did not overexpress PD-L1. Recovered grafts showed a decrease in T cell and NK cells in PD-L1-overexpressed cells [83]. Another recent study used a TALEN-mediated overexpression of PD-L1 on hiPSCs, which were then target-differentiated into beta-like cells. The authors reported that the overexpression of PD-L1 reduced the activation of β-cell destruction-inducing diabetogenic CD8 T cells, as observed through a significant decrease in interleukin-2 secretion. When combined with a CRISPR-based mutation of the B2M gene, there was a further reduction in interleukin-2 secretion, highlighting the possible advantage of combining multiple gene targets to enhance immunoprotection [65].

An important consideration of gene editing of hPSCs is the precision and safety of the gene editing process. In CRISPR-based gene editing, off-target editing could occur when DNA cleavage and repair happens at sites containing similar sequences as the target editing site. Though the chances of such inaccurate edits are relatively low, they could lead to significant safety issues such as the inhibition of tumor suppressor genes or the activation of oncogenes [84,85]. However, there are strategies designed to curb the chances of inaccurate off-target edits, such as improving the specificity of gene targeting by using a combination of sgRNA and high-fidelity Cas9 variants, or the use of multiple sgRNAs to target the same gene [86,87]. A crucial factor in the use of genetically modified β-cells to evade the immune system is the potential to be undetected by other immune mechanisms in the event of infections or tumor formations. Careful consideration and further studies need to be performed to validate the safety of these technologies as long-term solutions to treat diabetes.

A comparison of the various immune evasion strategies and their relative strengths and weaknesses are summarized in Table 1.

## 4. Stem Cell-Based Clinical Trials for the Treatment of Diabetes

As a potentially unlimited source of β-cells of autogenic origin, stem cells can reduce the dependence on organ donors and the associated complications for the treatment of diabetes, and this aspect of stem cells has been explored in several clinical trials in recent years. The source of stem cells that have been used in clinical trials is mesenchymal stem cells (MSCs), or pluripotent stem cells including hESCs or hiPSCs.

MSCs are adult multipotent stem cells capable of differentiating into osteoblasts (bone cells), chondrocytes (cartilage cells), myocytes (muscle cells), and adipocytes (fat cells). They are found in bone marrow, umbilical cord, or adipose tissues [91,92]. MSCs pose attractive qualities, such as an increased biosafety profile and a lower tumorgenicity risk, that render them an interesting choice for a potential diabetes treatment [93]. Additionally, they have regenerative properties, lack immunogenicity due to the absence of MHC class II, and have also been shown to support damaged islets [94,95,96]. Clinical trials have explored using MSCs in different settings to understand the best therapeutic method for the treatment of T1DM. The different hypotheses tested are (i) using undifferentiated MSCs to improve islet health and survival without differentiating into pancreatic progenitors, (ii) using MSC-derived pancreatic progenitors that differentiate into functional β-cells, and (iii) transplanting undifferentiated MSCs with the goal of in vivo transdifferentiation into functional β-cells [97,98]. One of the earliest clinical studies was developed to test the effect of autologous bone marrow MSCs using intravenous transplantation in T1DM patients. During the first year, an increased C-peptide response to mixed meal tolerance test (MMTT) was noted, and no apparent side effects were observed (NCT01068951) [99]. Another study assessed the long-term effects of an intravenous implantation of Wharton’s jelly-derived MSCs in newly diagnosed T1DM patients who were followed up for 21 months. The study concluded that patients with MSC treatment significantly improved HbA1c and C-peptide values as compared to pretreatment or control patients [100]. A study focused on the co-infusion of autologous adipose tissue-derived MSC-differentiated insulin-secreting cells and hematopoietic stem cells. Over a follow up period of over 31 months, the treatment improved mean C-peptide levels [101]. A recent pilot clinical study investigated the combined immunomodulatory effects of using MSCs and vitamin D in T1DM patients (NCT03920397). Patients were treated with intravenous MSC (allogenic) infusion, combined with oral cholecalciferol (vitamin D), and followed up for six months. An increase in basal C-peptide levels, which were stable for six months, was observed [102]. Another recent phase I/II clinical trial assessed the safety and efficacy of intravenous injection of MSCs in newly diagnosed T1DM patients who were followed up for at least one year post-transplant (NCT04078308). The study reported promising results where glycated hemoglobin (HbA1c) and C-peptide levels improved and shifted pro-inflammatory cytokines into anti-inflammatory cytokines. They suggested that an early transplantation of MSCs is favorable as compared to a late transplantation [96]. Several other clinical trials using MSCs have been completed with promising T1DM treatment observations [103,104,105].

Though MSC-based treatment strategies show promise in clinical settings, several weaknesses in the studies have been observed. The limited sample size of patients enrolled in most studies makes it difficult to draw concrete conclusions of the efficacy of MSC in T1DM treatment. Another valid point is that several clinical studies enrolled only patients who were recently diagnosed with T1DM. These studies reported high positive outcomes for the treatment and one study recommended early-stage T1DM intervention as compared to late-stage intervention. An interesting aspect of these clinical study designs is that the patients would be in the “honeymoon phase of diabetes”, wherein the patient would require only minimal insulin or have near-normal blood glucose levels without the need for insulin treatment. Successful treatment at this condition is not representative of the efficacy of the treatment on all T1DM patients. Clinical trials with a larger scope of patient enrollment with an increased sample size, diverse populations, and recent- and late-onset T1DM patients is necessary [96,106]. Although clinical studies of the use of MSC in T1DM treatment were inconclusive, excellent results have been observed for its use in T2DM treatment. A systematic review analyzing the results of 10 MSC-based clinical trials reported its effectiveness in improving β-cell function in T2DM. A positive outcome in stimulated C-peptide levels, HbA1c values, and the reduction in exogenous insulin requirement showed promise for the use of MSCs in β-cell therapy [107].

Human pluripotent stem cells are another promising route for the use of stem cells for the potential treatment of diabetes by utilizing hESCs and hiPSCs. The current clinical trials using hPSCs consider two schools of thought as to at what stage of β-cell differentiation cells can be transplanted. The first option is transplanting pancreatic progenitor cells that co-express PDX1 and NKX6.1. The co-expression of these two factors is critical in the generation of functional and monohormonal β-cells, as cells that do not co-express these transcription factors generally follow an alternative differentiation path resulting in non-functional, polyhormonal, or non-β-cells [108,109]. The first clinical trial using hESCs was performed in 2014 on 19 candidates by combining pancreatic progenitor cells [PEC-01] and an immunoprotective macroencapsulation device (PEC-Encap), produced by the clinical-stage regenerative medicine company, ViaCyte. The purpose of this study was to test whether the combination product, named VC-01, can be implanted subcutaneously in T1DM subjects and maintained safely for two years (NCT02239354). The macroencapsulation device was designed to prevent allogeneic and autoimmune rejection by protecting the pancreatic progenitor cells from the immune system, thereby excluding a dependence on immunosuppressive drugs. This was achieved by having a semipermeable membrane which allowed the diffusion of molecules but restricted the movement of cells. The study observed that the macroencapsulation device was affected by a foreign body response that prevented vascularization, leading to inconsistent cell survival, and no evidence of insulin secretion was found [97,110,111]. The findings from this study necessitated the need for an updated design for the encapsulation device to overcome immune response issues. ViaCyte initiated a second clinical trial in 2017 with an updated macroencapsulation device which was not immunoprotective but was designed to enable direct capillary vascular permeation into the encapsulation device. The system, named PEC-Direct, combined PEC-01 with the updated macroencapsulation device VC-02 (NCT03163511). In this study, 17 patients between the ages 22 and 57 with T1DM were recruited. Following subcutaneous transplantation, 63% of candidates responded to the treatment as presented by successful engraftment and increased insulin positive cells at 3–12 months post-transplantation. Approximately 35% of candidates showed the ability to secrete C-peptide 6 months post-transplantation. The observed side-effects were related to surgical implant/explant procedures or to immunosuppression [112]. A 1-year follow-up study on the recipients showed the absence of teratoma formation or severe graft-rejection. Patients showed increased fasting and glucose responsive C-peptide levels and developed mixed meal-stimulated C-peptide secretion. Also, explanted grafts contained mature β-cell phenotype and were immunoreactive for insulin, MAFA and islet amyloid polypeptide, suggesting post-transplantation maturation of the pancreatic progenitors [110]. Recently, an interim report was published for this clinical study which analyzed 1 year outcome for a study group that received 2–3-fold higher cell doses with an enhanced perforation pattern of the encapsulation device. It was observed that out of ten patients, three were able to achieve improved C-peptide levels and reduced insulin dosing from six months onwards. The authors attributed these positive changes to the formation of a larger β-cell mass due to a higher initial dose of transplanted cells. They also suggested that design changes in the encapsulation device might have improved capillary ingrowth in the implanted cell mass, thereby improving β-cell maturation [113]. Further optimization of the PEC-Direct device was done by ViaCyte in collaboration with CRISPR therapeutics to use genetically edited cells to circumvent immune responses and rejection. This was achieved by modifying several genes in pancreatic endoderm cells (PEC210A) using CRSIPR/Cas9 technology, including the deletion of β2-microglobulin gene and transgenic expression of PD-L1. In a 2022 clinical trial (NCT05210530), the combination product VCTX210A was used, which contained PEC210A cells encased in a durable and removable perforated encapsulation device designed to deliver and retain PEC210A cells. Findings from the 1-year study have yet to be published. In a 2019 clinical study, Viacyte revisited their PEC-encap technology in collaboration the material science company Gore to create a modified PEC-encap device which aims to eliminate the need for immunosuppression while promoting vascularization (NCT04678557). The two-year study with 49 candidates has recently reached its conclusion, and detailed results from this study are awaited [114].

The above clinical studies used pancreatic progenitors for transplantation as they are less likely to be affected by inflammation due to transplantation and post-transplantation maturation is expected to occur over time [55,97]. However, a second option of transplantable cells considered are hPSC-derived islet-like organoids. These organoids consist of fully differentiated and glucose responsive hPSC-derived β-cells and upon transplantation, they are quicker to achieve glycemic control as compared to pancreatic progenitors, thereby, making them an attractive source for the treatment of diabetes [115,116]. Currently, Vertex Pharmaceuticals has entered phase I/II clinical trial with hPSC-derived β-cells (VX-880) generated by Melton group (NCT04786262). The cells, which lack any encapsulation devices, are transplanted into the patients via the hepatic portal vein. Although the lack of encapsulation requires immune suppression of the patient, early reports of the treatment’s efficacy have been remarkably positive. Data collected 90 days post-transplant showed a 91% decrease in insulin requirement, mixed meal test-responsive elevation in circulating C-peptide and a reduction in HbA1c values [117]. Vertex is also actively recruiting candidates for a new phase I/II clinical trial in which patients are transplanted with hPSC-derived β-cells encapsulated in an immunoprotective device, thereby potentially bypassing immunosuppressive treatments (NCT05791201) [118]. Sigilon therapeutics, a subsidiary of the pharmaceutical company Eli Lilly and Company, has their encapsulated hiPSC-derived insulin-producing β-cells approaching clinical trials in the near future [97,112].

An important aspect of the successful transplantation of hPSC-derived pancreatic progenitors/β-cells is the cell delivery system employed. For a successful cell therapy treatment, cells should be both immunoprotected and have sufficient oxygen supply. Some of the hPSC-based clinical trials discussed above have used encapsulation devices to enhance cell survival and function. More methods of cell delivery have been developed and used in both preclinical and clinical trials using non-hPSC-based beta-cell transplantation. Alginate microencapsulation was the first cell delivery system used to protect transplanted β-cells. Clinical studies have used calcium/barium-alginate capsules to protect transplanted β-cells. Even though this encapsulation method prevented immune rejection, it was unable to significantly improve insulin release [119,120]. The biotech company Beta-02 Technologies has developed the Beta-air device, a “bioartificial pancreas” in which cells are placed in a slab of alginate and protected from the environment using a PTFE-based semipermeable membrane. A clinical trial using a subcutaneously transplanted “Beta-air” device produced small amounts of insulin, but not enough to reduce insulin dependency (NCT02064309). The disadvantage of this system was the need to continuously provide oxygen from an external source to maintain islet health and survival. An improved variant of the Beta-air device is being generated for hPSC-derived β-cells [51,97]. Cell Pouch^TM^, developed by the biotech company Sernova, is another cell delivery device being used in clinical trials for islet transplantation. Cell Pouch^TM^ is a polypropylene membrane-based rectangular microporous pouch with multiple parallel chambers filled with PTFE. After transplantation, the PTFE plugs are removed, and islets are introduced into the void. A 2018 clinical study utilizing this technology reported promising results in terms of vascularization and β-cell function (NCT03513939). Although this device currently does not prevent immunosuppression, Sernova is considering hydrogel capsules to protect the cells from the host immune system [121]. The Shielded Living Therapeutics^TM^ sphere by Sigilon is another cell delivery candidate that shows promise. The sphere contains an external coating of alginate modified with the triazole–thiomorphaline dioxide and an internal core of modified alginate matrix housing cell clusters. This design of the sphere provides it with immune protection that is lacking in most other devices. Sigilon used this device in a clinical trial (NCT04541628) for hemophilia patients, but the study was terminated following safety concerns [121]. In addition to the devices mentioned above that have achieved clinical testing, a number of other devices have been developed which are in the preclinical phase. TheraCyte^TM^ produced by TheraCyte, ceMED produced by Harvard-MIT Health Sciences and Technology, a bioengineered vascular bed by Technion, an oxygenation cell delivery device from Procyon Technologies, and an electrospun nanofibrous encapsulation device from Novo Nordisk are some examples of potential encapsulation devices that may reduce the dependency on immunosuppressive treatments in the future [58,122,123,124,125]. These novel technologies carry immense promise for the development of systems to transplant β-cells while holding the deleterious host immune responses at bay. These devices can be combined with hPSC- and MSC-based cell therapies for successful transplantation strategies to drive long-term treatment options with minimal side effects. A summary of recent clinical trials using stem cell derived pancreatic progenitors/β-cells is summarized in Table 2.

## 5. Conclusions and Future Directions

**Figure 2:**
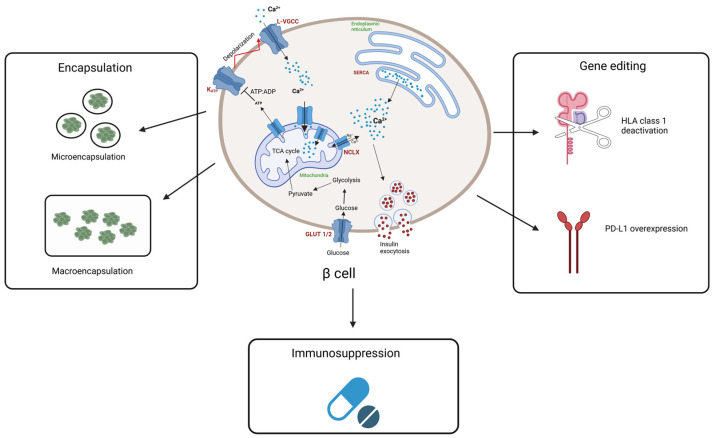
Schematic of the different approaches for the immunoprotection of hPSC-derived β-cell islets for transplantation. Encapsulation strategies involve microencapsulation and macroencapsulation, differentiated by the number of beta cells each method holds. Gene editing techniques include HLA class 1 deactivation using B2M gene knockout, and PD-L1 overexpression. Immunosuppression regimens follow several combinations of drugs to protect transplanted cells from host immune system. This illustration was created with “BioRender.com”.

In this review, we outlined the key challenges of β-cell islet transplantation with respect to graft-mediated host immune responses and emphasized the prospects of using hPSC-derived β-cells as a viable treatment strategy for diabetes. Immune-evasive hiPSC-derived β-cells hold promise as an autologous renewable cell source that can eliminate the necessity of cadaver islets for transplantation and can also reduce the dependence on immunosuppression regimens that compromise the host’s immune system and make them prone to infections and tumors. The advantages of combining stem cell technology with material sciences to create efficient encapsulation devices and gene editing tools to create immune evasive cells presents great excitement for the future of diabetes treatment. Great strides are being made to achieve the goal of generating hPSC-derived β-cells that mimic native β-cells. A summary of the different techniques explored to render immunoprotection to transplanted beta cells is shown in Figure 2.

Acute stress immediately after transplantation is a cause for the loss of a large percentage of transplanted cells. Hypoxia, inflammatory cytokines, and hyperglycemia can lead to apoptosis or dysfunction via ER stress responses [126]. Also, instant blood-mediated inflammatory reaction (IBMIR), an inflammatory response to contact with blood leading to platelet encapsulation of the transplanted cells, reduces the diffusion of nutrients into the cells [127]. Research is ongoing to address these concerns while considering hPSC-derived β-cell transplantation. A notable strategy to overcome these issues includes considering alternative transplantation sites such as intramuscular space and omentum [128,129]. However, these techniques have yet to circumvent transplantation stress effectively and further research needs to be performed to improve the safety of transplantation. Research to improve the process of creating enhanced functional hPSC-derived islets that would improve transplantation is also ongoing. Different hPSC differentiation protocols generate varying efficiencies of β-cells and efficiencies also vary between different hPSC cell lines, making the adoption of an ideal differentiation protocol for clinical use difficult [34]. Also, making this therapy accessible to a large number of patients would involve efficient methods to produce transplantable cells at large scales, while keeping production and treatment costs affordable. Current techniques for hPSC differentiation involve small-scale 2D plates or 3D bioreactors. Although current differentiation techniques can produce relatively high-quality β-cells, the same might not be possible to achieve at large-scale production. Further research is imperative to bridge knowledge gaps in the field to seamlessly transfer lab technologies to clinical scales. Consistent improvements are also being made to encapsulation devices to better protect transplanted cells. Numerous trials are underway that closely monitor the application of these technologies in a clinical setting. Although initial reports of these trials show promise in reducing insulin dependency in patients, a closer look into their safety and efficacy needs to be studied to promote their use as a long-term solution to cure diabetes.

## Figures and Tables

**Table 1:** A comparison of various immune evasion strategies, highlighting advantages and potential safety concerns.

Immune Evasion Strategy	Mechanism	Advantages	Safety Concerns	References
Immunosuppressive drug therapy	β-cells are protected by inducing a general suppression of the immune system (e.g., anti-rejection drugs)	- Broad suppression of immune activity can reduce both innate and adaptive immune responses. - Readily available and well-studied agents.	Risk of infection, autoreactivity, cancer	[25,88]
Encapsulation	β-cells are encapsulated in biocompatible materials that prevent immune cells from attacking them.	- Provides a physical barrier against immune cells and antibodies. - Reduces reliance on systemic immunosuppression.	Fibrotic responses, hypoxia, graft revascularization issues	[54,89,90]
Genetically engineered hPSC-derived β-cells	Modifies beta cells to express less immunogenicity or altered antigens (e.g., HLA knockout) to reduce immune recognition.	- Tailored to specific immune pathways, enabling precise modulation. - Can be combined with other therapies for synergistic effects.	Potential risks related to off-target effects or unintended gene modifications, which may lead to tumorigenesis or immune responses	[65,83]

**Table 2:** Stem cell based clinical trials for the treatment of diabetes.

Clinical Study ID	Start Year	Sponsor	Stem Cell Source	Status	Purpose of Study	Treatment Method	Reported Outcomes	Sample Size
NCT01068951	2010	Uppsala University Hospital, Sweden	MSCs	Completed	Evaluate the safety and efficacy of autologous MSCs in treatment of recently diagnosed patients with T1DM.	Intravenous transplantation.	Increased C-peptide response to MMTT.	20
NCT03920397	2015	Federal University of Rio de Janerio, Brazil	MSCs	Completed	Investigate the safety and efficacy of MSCs + daily cholecalciferol (VIT D) for 6 months in patients with recent-onset T1DM.	Allogeneic transplantation of adipose-derived MSCs with Vitamin D supplementation.	Increased basal C-peptide levels stable for 6 months.	30
NCT04078308	2015	Royan Institute, Iran	MSCs	Unknown	Examine the safety and efficacy of transplantation of MSCs in new-onset T1DM patients.	Intravenous transplantation of bone marrow-derived autologous MSCs.	Glycated hemoglobin (HbA1c) and C-peptide levels improved and shifted pro-inflammatory cytokines into anti-inflammatory cytokines.	21
NCT02239354	2014	Viacyte	hPSC	Completed	Test if VC-01^TM^ combination product can be implanted subcutaneously in subjects with T1DM and maintained safely for two years.	Tranplantation of pancreatic progenitor cells (PEC-01) in a macroencapsulation device (PEC-Encap).	The macroencapsulation device was impacted by the foreign body response, which hindered vascularization, resulting in inconsistent cell survival and the absence of insulin secretion.	19
NCT03163511	2017	Viacyte	hPSC	Completed	Test if VC-02^TM^ combination product can be implanted subcutaneously in subjects with T1DM and Hypoglycemia Unawareness and maintained safely for up to two years.	Transplantation of PEC-01 cells with the updated macroencapsulation device (VC-02).	63% of candidates showed a positive response to the treatment, demonstrated by successful engraftment and an increase in insulin-positive cells between 3 to 12 months after transplantation. Around 35% of candidates were able to secrete C-peptide 6 months post-transplant.	17
NCT04786262	2021	Vertex Pharmaceuticals Incorporated	hPSC	Recruiting	Evaluate the safety, tolerability, and efficacy of VX-880 cells infusion in participants with T1DM and impaired awareness of hypoglycemia and severe hypoglycemia.	Transplantation of hESC-derived β-cells (VX-880) via hepatic portal vein.	91% decrease in insulin requirement, mixed meal test-responsive elevation in circulating C-peptide, and a reduction in HbA1c values after 90 days.	17
NCT05791201	2023	Vertex Pharmaceuticals Incorporated	hPSC	Recruiting	Evaluate the safety, tolerability, and efficacy of VX-264 in participants with T1DM.	Transplantation of hESC-derived β-cells encapsulated in an immunoprotective device.	N/A	17
NCT04678557	2019	Viacyte	hPSC	Terminated	Evaluate an experimental combination product, cell replacement therapy intended to provide a functional cure to subjects with T1DM.	Transplantation of PEC-01 cells with encapsulation device (VC-01).	Insufficient functional product engraftment.	31
NCT05210530	2022	Viacyte, CRISPR Therapeutics AG	hPSC	Completed	Evaluate the safety and tolerability of VCTX210A combination product in patients with T1DM.	Transplantation of CRISPR-based genetically modified pancreatic endoderm cells (PEC210A) encased in a durable and removable perforated encapsulation device designed to deliver and retain PEC210A cells.	N/A	7

## Data Availability

No new data were created or analyzed in this study. Data sharing is not applicable to this article.

## Abbreviations

The following abbreviations are used in this manuscript:

T1DM	Type 1 diabetes mellitus
T2DM	Type 2 diabetes mellitus
hPSC	human pluripotent stem cells
IDF	International Diabetes Federation
ADA	American Diabetes Association
GDM	gestational diabetes mellitus
MODY	maturity-onset diabetes of the young
hESC	human embryonic stem cells
ER	Endoplasmic reticulum
hiPSC	human induced pluripotent stem cells
TCR	T cell receptor
MHC	Major histocompatibility complexes
APC	Antigen presenting cells
GSIS	glucose-stimulated insulin secretion
NOD-scid	non-obese diabeteic/severe combined immunodeficiency
HLA	human leukocyte antigens
PD-L1	Programmed death-ligand 1
PD-1	Programmed death 1
DSB	Double-stranded DNA break
ZFN	Zinc finger nucleases
TALEN	Transcription activator-like effector nuclease
CRISPR	Clustered Regularly interspaced-Short Palindromic Repeats
TALEs	Transcription activator-like effectors
sgRNA	short noncoding guide RNAs
Cas9	CRISPR-associated protein 9
B2M	Beta 2 microglobulin
KO	knockout
MSC	mesenchymal stem cells
MMTT	mixed meal tolerance test
IBMIR	Instant blood-mediated inflammatory reaction
